# Matrix Metalloproteinases as Regulators of Periodontal Inflammation

**DOI:** 10.3390/ijms18020440

**Published:** 2017-02-17

**Authors:** Cavalla Franco, Hernández-Ríos Patricia, Sorsa Timo, Biguetti Claudia, Hernández Marcela

**Affiliations:** 1 Department of Conservative Dentistry, School of Dentistry, Universidad de Chile, Santiago 8380492, Chile; francocavalla@gmail.com (C.F.); phernandezrios@gmail.com (H.-R.P.); 2 Department of Biological Sciences, School of Dentistry of Bauru, University of São Paulo (FOB/USP), Bauru, São Paulo 17012-901, Brazil; biguetti@usp.br; 3 Center for Craniofacial Research, University of Texas School of Dentistry at Houston, Houston, TX 77054, USA; 4 Department of Oral and Maxillofacial Diseases, Helsinki University and Helsinki University Central Hospital, Helsinki 00290, Finland; timo.sorsa@helsinki.fi; 5 Division of Periodontology, Department of Dental Medicine, Karolinska Institutet, Huddinge 14183, Sweden; 6 Laboratory of Periodontal Biology, Faculty of Dentistry, Universidad de Chile, Santiago 8380492, Chile; 7 Dentistry Unit, Faculty of Health Sciences, Universidad Autónoma de Chile, Santiago 8910060, Chile

**Keywords:** MMPs, chronic periodontitis, periodontal inflammation regulation, modulation

## Abstract

Periodontitis are infectious diseases characterized by immune-mediated destruction of periodontal supporting tissues and tooth loss. Matrix metalloproteinases (MMPs) are key proteases involved in destructive periodontal diseases. The study and interest in MMP has been fuelled by emerging evidence demonstrating the broad spectrum of molecules that can be cleaved by them and the myriad of biological processes that they can potentially regulate. The huge complexity of MMP functions within the ‘protease web’ is crucial for many physiologic and pathologic processes, including immunity, inflammation, bone resorption, and wound healing. Evidence points out that MMPs assemble in activation cascades and besides their classical extracellular matrix substrates, they cleave several signalling molecules—such as cytokines, chemokines, and growth factors, among others—regulating their biological functions and/or bioavailability during periodontal diseases. In this review, we provide an overview of emerging evidence of MMPs as regulators of periodontal inflammation.

## 1. Introduction

Periodontal diseases are common chronic pathologies worldwide that affect periodontal tissues, which include the gingiva, periodontal ligament, radicular cementum, and alveolar bone. Bacterial–host interactions at the biofilm–periodontium interface will initially trigger gingival inflammation, resulting in gingivitis. Over time, it can progress to the immune-mediated loss of the periodontal supporting tissues, including alveolar bone, determining the destructive character of the disease [1,2,3]. Besides representing the first cause of tooth loss in adults, periodontitis contributes to enhanced susceptibility to other systemic diseases, including cardiovascular diseases, diabetes [4], and lung inflammation, among others [5,6]. 

Matrix metalloproteinases (MMPs) are key proteases involved in destructive periodontal diseases [7,8]. The 23 existing MMPs are zinc-dependent endopeptidases that belong to the metalloproteinase superfamily [9]. The basic structure of MMPs is composed of an auto-inhibitory prodomain for enzymatic latency, the catalytic domain, and the C-terminal hemopexin-like domain, often involved in the recognition and positioning of MMP substrates. MMP activity is tightly regulated through gene expression, proenzyme activation, and enzyme inhibition by endogenous inhibitors, such as tissue inhibitors of MMPs (TIMPs). The four human TIMPs [1,2,3,4] are broad-spectrum MMP inhibitors, but differ among their specificity for MMPs. Consequently, MMPs can be found in periodontal tissues as pro-forms, active forms, complexed species, fragmented species, and cell-bound MMPs [10,11].

MMPs were traditionally regarded to degrade extracellular matrix components and grouped according to their substrate specificity in collagenases, gelatinases, stromelysins, matrilysins, and membrane type (MT) MMPs [12]. Because type I collagen represents the bulk component of periodontal extracellular matrix, special attention has been paid to collagenases—particularly MMP-8 and MMP-13- and gelatinases—MMP-2, and MMP-9- in periodontitis. In fact, MMP-8 (or collagenase 2) currently represents one of the most promising biomarkers for periodontitis in oral fluids [6,11]. Nevertheless, the concept of extracellular matrix-degrading enzymes represents an incomplete vision of MMP biology; on the one hand, limited proteolysis does not necessarily mean substrate degradation, but transformation with either gaining or loss of function; on the other hand, MMP substrates are not limited to the extracellular matrix components. In fact, a more updated concept of MMPs releasing cryptic functions of matrix proteins and processing of bioactive non-matrix substrates has emerged [13,14]. 

Overall, the huge complexity of MMP functions positions them into the current concept of the “protease web”. This implies that far from acting alone, these proteases assemble in cascades of zymogens, activators, inhibitors, and other bioactive substrates; in which proteolytic cleavage modulates the potential of the entire system with far-reaching effects, affecting other MMPs, as well as their substrates and functions. Given their multifaceted properties, MMPs regulate cell proliferation, adhesion, migration [15], growth factor bioavailability, chemotaxis, and signaling; and they are crucial for angiogenesis, vasodilation, tumorigenesis [16], metastasis [17], immunity, inflammation, and wound healing [13].

Despite its infectious etiology, the loss of periodontal supporting tissues during periodontitis is considered as an ultimate consequence of the host’s immune response. MMPs have been proposed as master regulators of inflammation, through proteolysis of chemokines, growth factors, receptors and their binding proteins, proteases, protease inhibitors, as well as intracellular multifunctional proteins, resulting in pro- or anti-inflammatory functions leading to either tissue homeostasis or pathology. In fact, MMPs have been associated with a wide range of inflammatory disorders [18]. MMP upregulation and downregulation in response to inflammatory mediators, such as cytokines and chemokines has been long demonstrated [19,20], but the existence of feedback mechanisms has been highlighted in recent years [9]. Migration of immune cells by processing extracellular matrix; stimulation and cessation of immune-cell recruitment by processing chemokines and other chemotactic molecules; exacerbation and resolution of inflammatory responses by processing complement, cytokines, and other non-matrix bioactive molecules have been reported [9,12,21,22,23,24,25]. In contrast to the vast array of literature assessing the potential of collagenolytic MMPs as periodontal disease biomarkers and effectors of collagen matrix degradation, few investigations have targeted their regulatory roles within the frame of the protease web of periodontitis. In this review, we provide an overview of MMP roles as regulators of periodontal inflammation.

## 2. Matrix Metalloproteinase Activation Cascades

Matrix metalloproteinases are synthesized as pro-enzymes, inactive forms resulting from the interaction between the cysteine residue of the prodomain and the zinc ion of the catalytic site. The disruption of this interaction by chemical modification or proteolytic removal of the enzyme’s prodomain results in enzyme activation, a mechanism known as the ‘cysteine switch’ [26]. Several MMPs have been detected in their different enzymatic forms in gingival tissue, gingival crevicular fluid (GFC), saliva, and mouth rinse, with MMP-8, MMP-13, and MMP-9 as the main proteases involved periodontal disease destruction [6,11,25,27].

MMP-8 and MMP-9 are the most abundant MMPs in periodontal tissues reflecting periodontal disease severity, progression, and treatment response [28]. They are secreted by infiltrating polymorphonuclear leukocytes, but also macrophages, plasma cells, and resident cells—such as fibroblasts, endothelial cells, keratinocytes and bone cells [29]. MMP-13 is a collagenase that has been detected in fibroblasts, macrophages, osteoblasts, plasma cells, and gingival epithelial cells. Though less abundant, MMP-13 has been implicated in periodontal soft tissue destruction and, along with MMP-9, it has been involved in alveolar bone resorption and periodontal tissue breakdown [25,30]. The mechanisms regulating MMP activation may vary depending on the specific tissue and disease microenvironment. During periodontitis progression, these MMPs can be activated by independent or co-operative cascades involving pathogen and host proteases [7,31,32,33].

**Figure 1:**
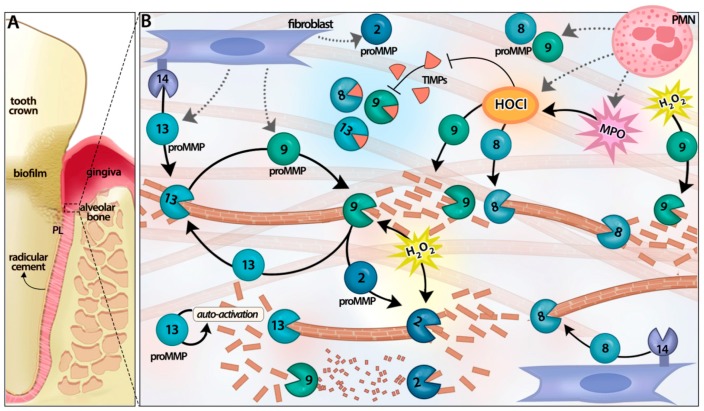
Matrix metalloproteinase (MMP) activation cascades in connective tissue catabolism during periodontitis. Full and partial circles with their respective number represent latent and activated specific MMPs. Brown T bars represent collagen fibers. (**A**) Tooth and its supporting structures: radicular cement, periodontal ligament (PL) and alveolar bone; (**B**) Membrane bounded MMP-14 activates proMMP-13 to degrade type I collagen, which constitutes the bulk component of radicular cement, periodontal ligament (PL) and alveolar bone extracellular matrix; MMP-13 activates proMMP-9, which in turn might activate proMMP-2 and proMMP-13. MMP-2 and MMP-9 further process gelatin resulting from collagenase activity. MMP-13 can undergo auto-activation by self-proteolysis. Reactive oxygen species, such as HOCl and H_2_O_2_, from phagocytes can also modify the proteases/anti-proteases balance, activating latent MMPs and inactivating the tissue inhibitor of MMPs (TIMP)-1. MPO, myeloperoxidase.

MMP proteolytic cascades can lead to widespread periodontal tissue destruction due to cooperative MMP activation and might represent an interesting target for diagnostics and therapy [32] (Figure 1). MMP-13 is able to induce proMMP-9 activation and MMP-13 auto-activation by self-proteolysis in vitro [12]. A previous study from our group reported that MMP-13 induces proMMP-9 activation in gingival tissues from periodontitis patients [32]. MMP-9, in turn, can activate proMMP-2 and proMMP-13 in vitro [12,34]. Collagenase levels of MMP-14, MMP-8, and MMP-13, have been correlated in chronic periodontitis patients, which suggests either a co-operative role and/or the potential settlement of collagenase activation cascades [35]. MMP-14 can activate MMP-8 and -13 in vitro, as well as MMP-2, and has been correlated in vivo with active MMP-13 in periodontitis sites [21,31,36]. Altogether, these mechanisms could enhance periodontal tissue breakdown and consequent chronic periodontitis progression [32].

Additionally, oxidative non-proteolytic MMP activation seems to be pivotal in periodontal inflammation. Reactive oxygen species (ROS) are able to induce the activation of the key MMPs in periodontal tissues, through direct enzyme oxidation [37], although indirect mechanisms cannot be precluded. Neutrophil degranulation is stimulated by periodontopathogenic bacteria, their virulence factors, cytokines, and prostaglandins and results in the release of myeloperoxidase (MPO) from their primary granules [35,38,39,40]. MPO catalyzes the conversion of the potent oxidant hypochlorous acid (HOCl), and besides its antimicrobial activity, it plays an important role in the regulation of connective tissue catabolism and degradation, through modifying the proteases/anti-proteases balance [40]. In fact, HOCl can rapidly activate proMMP-8 and proMMP-9 depending on the oxidant/enzyme molar ratio [37,41,42]. Ex vivo evidence from progressive chronic periodontitis in humans supports a role for MPO in direct activation of latent MMP-8 and MMP-9, and the inactivation of TIMP-1 [31,43,44]. MPO levels repeatedly correlate not only with total MMP-8 levels, but also with its active isoenzyme forms [31,45]; and both MPO and MMP-8 show high accuracy for specific site-diagnosis of chronic periodontitis versus gingivitis and healthy sites [35]. These findings suggest that MPO might play a critical role enhancing MMP-8 activation during periodontal disease progression. 

ROS-mediated MMP activation is also supported in different cell culture systems. Oxidative stress increases extracellular matrix turnover mediated by MMP-2, -9, and -13 in fibroblastic and tumor cells [46,47,48]. Low concentrations of ROS also play a role in cell signal transduction through the presence of redox-sensitive cysteines activating transcription factors, such as nuclear factor kappa B (NFκB), and Ca^2+^ membrane channels [47,49]. Peroxide stimulus can increase the activity of MMP-2 and -9 in fibroblastic cell lines (TIG-7), whereas bacterial-induced activation of MMP-2 can be blocked by an NFĸB inhibitor, supporting the involvement of NFĸB signaling pathway [50]. Recently, our group reported that non-toxic low doses of H_2_O_2_ induce MMP-2 and MMP-9 activation involving a cross talk between NFκB and intracellular calcium signals in periodontal fibroblast primary cultures [51], evidencing intracellular oxidative-induced signaling for MMP activation.

## 3. MMP-Mediated Regulation of Inflammatory Response

**Figure 2:**
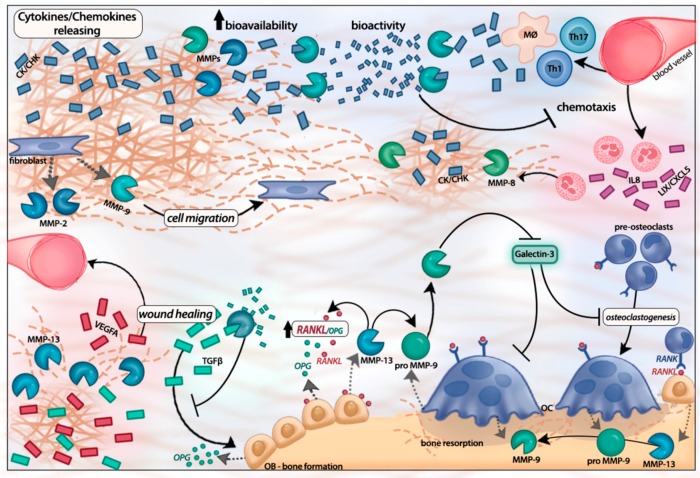
MMPs as regulators of periodontal inflammation. MMPs release cytokines/chemokines from the ECM, increasing their bioavailability and modifying their bioactivity. Conversely, MMPs can also cleave cytokines/chemokines generating truncated products that can act as competitive antagonists. The relative abundance of chemokines and cytokines favoring the infiltration of neutrophils (e.g., CXC motif chemokine ligand 8, a.k.a. CXCL8; LIX/CXCL5), macrophages (e.g., CC motif chemokine ligand 2, a.k.a. CCL2), and Th1/Th17 lymphocytes (e.g., CC motif chemokine ligand 20, a.k.a. CCL20) is determinant in the inflammatory destruction of periodontal tissues. MMPs can regulate wound healing, releasing angiogenic factors—such as VEGF A—from the extracellular matrix or through modifying transforming growth factor (TGF)-β1 signaling. MMPs also regulate bone resorption. MMP-13 activates osteoclast-secreted proMMP-9, inactivates galectin-3, an inhibitor of osteoclastogenesis, and favors receptor activator of nuclear factor-κB ligand (RANKL) and TGF-β1 signaling. CK/CC = cytokines/chemokines; OB = osteoblast; OC = osteoclast; MØ = macrophage; Th = T helper CD4+ lymphocytes.

Several signaling molecules such as cytokines, chemokines, and growth factors can be processed by active MMPs, thus regulating their biological functions and/or bio availability [18,36,52,53,54,55,56]. Cytokines are key modulators of cellular responses during periodontal inflammation and, upon coupling with their cognate receptors in their cellular targets, they induce intracellular signaling and modify gene expression. Likewise, chemokines are small signaling molecules responsible for the regulated trafficking of specific cell types into the inflamed tissues [57]. The balance and relative abundance of chemokines and cytokines is the ultimate responsible for the tissue changes during the progression of periodontitis and the tilting of their balance could determine the duration, intensity, and character of the response [58,59]. Depending on the relative abundance and kinetics of the expression of these molecular signals the response to periodontal infection can range from subclinical inflammation to destructive periodontitis [60]. The MMP regulatory mechanisms in periodontal inflammation are represented in Figure 2.

The persistent secretion of pro inflammatory Th1 cytokines in the periodontal tissues, such as tumor necrosis factor alpha (TNFα), interleukin (IL)-1β, IL-6, IL-12, and interferon gamma (IFN-γ); Th17 cytokines, such as interleukin IL-17, together with low levels of regulatory cytokines (Treg), such as IL-10, and transforming growth factor beta 1 (TGF-β1), has been associated with continued inflammation and the destruction of the supporting structures of teeth [61]. Similarly, the relative abundance of chemokines favoring the infiltration of neutrophils (e.g., CXC motif chemokine ligand 8, a.k.a. CXCL8), macrophages (e.g., CC motif chemokine ligand 2, a.k.a. CCL2), and Th1/Th17 lymphocytes (e.g., CC motif chemokine ligand 20, a.k.a. CCL20) has been associated with the inflammatory destruction of periodontal tissues. Notably, most of the former signaling molecules are targets for MMP cleavage and hence their biological activity could be regulated by MMPs [62].

In a recent article, we demonstrated that MMP activity decreased the secreted levels of IL-6, while at the same time it was responsible for increasing the secreted levels of CXC motif chemokine ligand 12 (CXCL12) and vascular endothelial growth factor A (VEGF A), and reduced cell migration in an in vitro system of human periodontal ligament fibroblasts [63]. The target-specific effect of MMP activity highlights the complexity of the networks that are at play during the inflammatory process. Arguably, the increased levels of CXCL12 and VEGF could be the result of the proteolytic modification either of the matrix or the cell surface and the liberation of trapped molecules to the culture media.

Even though CXCL12 has been described as a substrate for MMP-2 and MMP-9 [55,64], the activity of these MMPs increased its soluble levels in our model. Nevertheless, it is worth noting that the difference between the MMP-cleaved and native forms of CXCL12 could be indiscernible depending on the coupling site of the antibody used for the quantification. In this later case, it is possible that the increased levels do not necessarily mean increased biological activity, since the MMP-cleaved form of CXCL12 is significantly less active than the native form [64]. Remarkably, the gelatinases MMP-2 and MMP-9 appear to be particularly prone to cleave CXC motif chemokines, conferring them a formerly unrecognized key role in the regulation of cell trafficking in various pathologies characterized by uncontrolled inflammatory response, such as periodontitis, rheumatoid arthritis, and cancer [65].

Evidence from experimental periodontitis models shows a more severe disease phenotype in MMP-8 null mice infected with *Porphyromonas gingivalis* than their wild type counterparts, along with significantly lower levels of lipopolysaccharide (LPS)-induced CXC chemokine (LIX/CXCL5) and reduced neutrophil infiltration. These features suggest an impaired LIX/CXCL5-mediated neutrophil chemotaxis to the periodontal-biofilm interface, where neutrophils represent the first line of defense against periodontal pathogens [36,66]. In line with this, several studies support a role for MMP-8 in neutrophil trafficking and apoptosis in different inflammation models, such as wound healing and TNFα-induced lethal hepatitis [18,56].

Additionally, several MMPs can cleave CC motif chemokines, producing truncated products that act as potent antagonist of their cognate CC chemokine receptors. For example, CC chemokine ligand 7 (a.k.a. MCP-3) can be cleaved by MMP-2, MT1-MMP, MMP-1, MMP-13, and MMP-3 [67]. Interestingly, CC motif chemokines have a fundamental role in the recruitment of monocytes from the circulation to the periodontal tissues during the progression of periodontitis, and CCL7/MCP-3 has particularly been shown to be selectively upregulated in progressive sites from chronic periodontitis patients [68]. Since MMPs are upregulated during periodontal inflammation, the proteolytic inactivation of CC motif chemokines could represent a regulatory feedback mechanism to prevent uncontrolled monocyte infiltration, contributing to the resolution of inflammation [67].

New in vivo experimental evidence also demonstrates that even during the normal immune response to infection, MMPs can exert regulatory roles modulating the levels and bio-availability of cytokines. In a murine model of *Staphylococcus aureus*-induced septic arthritis it was demonstrated that the inhibition of MMP-2 activity resulted in significantly decreased serum levels of the pro inflammatory cytokines TNFα, IL-1β, IL-6, IL-12, IFN-γ and increased levels of the anti-inflammatory cytokine IL-10 [69]. This evidence emphasizes that the regulatory roles of MMPs can lead to profound effects that tilt the balance towards resolution or perpetuation of the inflammatory response, and that these changes are strong enough to be reflected in serum levels of inflammatory biomarkers. 

MMPs are also involved in wound healing. Recently, our group reported faster wound closure in lingual mucosa along with higher TGF-β1 levels in MMP-8 null mice compared to their wild type controls. MMP-8 showed to degrade TGF-β1 and alter its signaling in vitro, explaining its lower levels in wild-type animals [70]. Similarly, MMP-9 activity was shown to downregulate TGF-β1 protein levels in breast cancer cells exposed to tamoxifen [71,72]. Conversely, dermal wound closure was slower in MMP-8 null mouse model and inflammatory response was altered with a delay in neutrophil infiltration and persistent inflammation at a later phase. Reduced levels of active TGF-β1 and increased Smad-2 and Smad-3 signaling were demonstrated in MMP-8^−/−^ when compared to wild type animals, whereas expression of the CXC motif chemokine ligand 1 (CXCL1) and CXC motif chemokine ligand 2 (CXCL2) was delayed, which might account for the persistent inflammation observed in the model [56]. These results highlight the complexity of the regulatory roles of MMPs, which might also depend on the tissue specific microenvironment.

MMPs can modulate bone resorption through osteoclast activation and differentiation, besides direct bone collagen matrix degradation. In vivo and in vitro studies in breast cancer bone metastasis support that MMP-13 is required for the differentiation of pre-osteoclasts, osteoclast activation, and osteolysis. Several mechanisms have been reported, which include the activation of osteoclast-secreted proMMP-9, which further digests denatured collagen derived from MMP-13 activity [73]; cleaving of galectin-3, a known inhibitor of osteoclastogenesis expressed in the osteoclast surface, that results in the abrogation of its inhibitory effect; and by regulating the receptor activator of nuclear factor-κB ligand (RANKL)/osteoprotegerin (OPG) axis, favoring RANKL and TGF-β1 signaling via phospho-Smad-2 [74,75].

MMP activity is also involved in the regulation of cytokine-independent pathways, such as nerve/glial antigen 2 (NG2)-mediated apoptosis. NG2 is a recently characterized receptor that induces apoptosis in cells that have lost their matrix contacts (anoikis) [76]. In an in vitro model of periodontal ligament fibroblasts, it has been demonstrated that the cleavage of NG2 by MMP-13 is essential to terminate the signaling that leads to apoptosis and its inhibition results in increased apoptosis, coinciding with diminished soluble levels of NG2. This evidence suggests that NG2 needs to be cleaved and released from the cell membrane by MMP-13 in order to terminate the pro apoptotic signaling [77].

Interestingly, most of the activating signals for MMPs and pro inflammatory cytokines/chemokines are common, as well as the signaling pathways that lead to increased transcriptional activity of their genes. Still, in some cases, there are regulatory networks in place in which the signaling of a specific cytokine leads to the negative regulation of the transcription of *MMP* genes. One such example is the negative regulation of MMP-3 by IL-4. MMP-3 has also been involved in periodontal matrix degradation and it has been long known that its transcription is controlled by IL-1β-mediated activation of the transcription factor activating protein 1 (AP-1). A recent report demonstrated that IL-4 signaling exerts an inhibitory effect on *MMP3* gene transcription, and that this is at least partially mediated by the induction of an alternative assembly of the component dimers of the multiprotein complex AP-1, decreasing its inherent affinity for the promoter site of the *MMP3* gene [78]. In yet another example of the interplay of MMPs and cytokines, it has been recently reported that MMP-12 regulates the levels of the antiviral cytokine IFN-α by cleaving off the IFN-α receptor 2 binding site of systemic IFN-α, but also acting as a transcription factor, translocating into the nucleus and directly binding to the NFKBIA promoter, driving transcription and increasing intracellular levels of IκBα, which is the main protein responsible for IFN-α extracellular export [79].

This novel role of MMPs as regulators of transcription has also been demonstrated in non-immune pathways, such as of cell growth and cell metabolism. For example, MMP-3 has been localized in the nuclei of chondrocytes and it has been demonstrated that it can interact with the transcription enhancer dominant in chondrocytes (TRENDIC) in the promoter region of the connective tissue growth factor (*CCN2/CTGF*) gene and that the overexpression or addition of recombinant MMP-3 increases the transcriptional activity of *CCN2/CTGF* [80].

An extensive body of evidence has been published regarding the putative association of polymorphism affecting the function of diverse MMPs and the risk of periodontitis. Despite some conflicting reports, recently published systematic reviews including meta-analysis seem to establish that an association indeed exists. A meta-analysis involving in excess of 6000 individuals established that *MMP-9*-753 C/T polymorphism reduced the risk of chronic periodontitis, while *MMP-3*-1171 5A/6A and *MMP-8*-799 C/T polymorphisms increased the risk of chronic periodontitis [81]. Similarly, another meta-analysis including almost 3000 subjects demonstrated that *MMP-1*-1607 1G/2G (rs1799750) polymorphism was associated with chronic periodontitis, especially with the severity of the disease condition [82]. Even though the specific underling mechanism to the association between *MMP* genepolymorphisms and periodontitis is still unclear, it probably includes all the regulatory features discussed in the previous paragraphs.

## 4. Concluding Remarks

Increasing evidence suggests that MMPs play a much more significant role in inflammation and immune response regulation than previously recognized. In periodontally inflamed sites, they are capable of participating in cross-activation and auto-activation cascades, as well as regulating the availability of many inflammatory signaling molecules. The bidirectional regulation of MMPs and cytokine/chemokine levels appears to be tightly controlled, but our current understanding of the process is far from complete. As previously discussed, there are profound interactions between cytokines and MMP, with mutual regulatory features at all levels, from transcription to proteolytic modulation of biological functions. Depending on the stimulus and the local environment, MMPs could increase or decrease the bioavailability of signaling molecules by several different and often complementary mechanisms that can result in widespread periodontal supporting tissue loss and sustained inflammation.
